# Food Supplements and Well-Being: A Pilot Investigation in the General Practitioner Office of the Veneto Region

**DOI:** 10.3390/healthcare14091189

**Published:** 2026-04-29

**Authors:** Raffaele Pezzani, Susi Barollo, Sara Vitalini, Francesco Trevisan

**Affiliations:** 1 Accademia Italiana di Fitoterapia, Via Ugo la Malfa, 24, 25100 Brescia, Italy; 2 Phytotherapy Laboratory, Department of Medicine (DIMED), University of Padova, Via Ospedale 105, 35128 Padova, Italy; susi.barollo@unipd.it; 3 Department of Biomedical, Surgical and Dental Sciences, University of Milan, Via della Commenda 10, 20122 Milano, Italy; sara.vitalini@unimi.it; 4 ULSS7 Pedemontana, Primary Care Unit, Via Monte Sisemol 2, 36012 Asiago, Italy; francesco.trevisan@univr.it

**Keywords:** food supplements, Veneto, general practitioner, psychophysical health, well-being

## Abstract

**Highlights:**

**What are the main findings?**

- High prevalence of food supplement (FS) use (73.5%) among GP patients in Veneto, Italy, primarily for general well-being, immune support, and energy, with vitamins, minerals, and probiotics being most consumed.
- Significant gap in patient-physician communication: only 57.4% of FS users informed their GP, while 66.3% self-prescribed, raising potential safety concerns regarding drug-supplement interactions and inadequate professional supervision.

**What are the implications of the main findings?**

- Lower mental health scores (MCS-12) compared to the Italian population average suggest psychological distress among GP patients, highlighting the need for integrated mental health support in primary care settings.
- Urgent need for enhanced health education and professional training to ensure safer FS use, improve patient-physician communication, and address the misconception that “natural equals harmless”.

**Abstract:**

Background: The use of food supplements (FS) is rapidly increasing, particularly in Italy, which leads the European market. This trend is driven by various factors, including the pursuit of physical well-being, the influence of advertising, and concerns about disease prevention. This exploratory pilot descriptive study aimed to characterize FS use among patients attending general practitioner (GP) offices and examine potential patterns with psychophysical well-being. Methods: Two questionnaires were administered to participants: one on FS use and another on physical and mental health (SF-12 questionnaire). General information and anthropometric characteristics were also collected. Results: 230 questionnaires on FS use and 192 on psychophysical well-being were analyzed. The majority of participants (73.5%) reported using FS, primarily for general well-being (21.0%), immune system support (12.2%), and increased energy (11.4%). The most commonly consumed FS were vitamins (19.4%), minerals (16.9%), and probiotics (15.7%). Only 57.4% of patients reported informing their doctor about FS use, while 66.3% engaged in self-prescription. The SF-12 questionnaire revealed lower mental health scores (mood, energy, anxiety, and depression) in the studied population, while physical health remained unaffected. Importantly, no significant associations were observed between FS use and either physical or mental health scores, suggesting these patterns are independent of supplement consumption. Conclusions: FS use is prevalent among patients attending GP offices. The observed decrease in mental health scores may indicate psychological distress though this pattern was not associated with FS consumption. Given the exploratory nature of this study, findings should be interpreted with caution. This study highlights the need for improved health education and professional training to promote safer and more informed FS use. Further research is required to expand upon these initial findings.

## 1. Introduction

In recent decades, the use of food supplements (FS) has evolved into a widespread phenomenon, encompassing an increasingly large portion of the population [1,2]. This trend is driven by diverse factors, including the desire to enhance physical well-being, the influence of advertising, the pursuit of optimal sports performance, and concerns about nutritional deficiencies or disease prevention [1,3]. Given the sustained increase in FS consumption documented over the past decade, the role of physicians, actively engaged or inadvertently implicated, is becoming increasingly significant [2]. Italy leads the European FS market with a 27% share (compared to Germany’s 18% and France’s 8%), valued at approximately 4 billion euros [4]. A recent Censis study reported that 32 million Italians use FS, with 18 million consuming them daily or several times a week, and over 4 million using them a few times a month [5]. Consumers are attracted to FS by promises of health improvement and well-being benefits. This positive perception is often amplified by advertising campaigns that emphasize benefits while frequently neglecting to provide adequate information on potential risks or the importance for medical supervision. Physicians often serve as the primary point of reference for patients seeking advice on FS use, with an estimated 82.4% of Italian FS users receiving recommendations from healthcare professionals (GPs, specialists, or pharmacists). The remaining 17.6% obtain advice from family, friends, the internet, TV, or magazines [5]. The relationship between FS use and psychophysical well-being is theoretically plausible yet empirically complex. Nutritional deficiencies are known to impair both physical functioning and mental health, and targeted supplementation may address such gaps in specific populations [6]; however, evidence for general well-being benefits in non-deficient individuals remains mixed and inconclusive. While specific supplementation has demonstrated benefit in well-defined clinical contexts (e.g., folate in pregnancy, vitamin D in deficiency states), broader claims lack consistent scientific support [7]. In Italy, FS are regulated as foodstuffs under Legislative Decree 169/2004 (EU Directive 2002/46/EC), requiring only Ministry of Health notification rather than pre-market efficacy or safety demonstration, creating a permissive environment for uncritical consumer adoption. Furthermore, FS use has been paradoxically associated with both greater health-seeking behavior and increased self-medication with reduced physician consultation, with psychosocial factors—including health anxiety and post-pandemic vulnerability—representing additional drivers of supplement uptake [8,9]. Despite this, robust empirical data linking FS consumption to health-related quality of life (HRQoL) in primary care settings remain scarce, justifying the present exploratory investigation using the validated SF-12 instrument [10,11].

However, evidence supporting the efficacy of many FS for general health outcomes remains limited or inconclusive, and potential risks including drug interactions and adverse effects are often underappreciated by consumers.

Despite Italy leading the European FS market, data on FS use patterns specifically in primary care settings and their relationship with psychophysical well-being remain scarce. Unlike general population surveys or studies focused on specific subpopulations such as university students [12], no Italian primary care study has systematically examined the association between FS consumption and HRQoL using a validated instrument. This pilot cross-sectional study addressed three research questions: (1) what is the prevalence and pattern of FS use among adults attending GP offices in the Veneto region, including types, frequency, and motivations for use; (2) what are the primary information sources and recommendation pathways influencing FS consumption in this population; (3) to determine if FS use is associated with physical and mental health-related quality of life, as measured by the SF-12, in primary care attendees. By addressing these questions, this study aims to contribute descriptive evidence on FS use in an Italian primary care context, where such data remain limited.

## 2. Materials and Methods

### 2.1. Study Population

The study was performed in accordance with the Declaration of Helsinki and its current amendments, and all subjects provided written informed consent prior to participating in the study. For this observational study no ethical approval was necessary for the Italian legislation [13].

The Veneto region was selected as the study setting primarily for logistical reasons, as the research (September 2024 to April 2025). The inclusion criteria were: age > 18 years, living in Veneto Region, willingness to participate to the study. The exclusion criteria comprised any physical, cognitive, or clinical condition that could prevent accurate completion of the questionnaires.

### 2.2. Questionnaires

Paper-based cross-sectional surveys were conducted among population attending the GP offices in the Veneto region, involving 9 GP offices in both urban and rural settings. Potential participants were informed about the study through paper notices displayed prominently in the waiting rooms of participating GP offices. These notices explained the study purpose, the voluntary and anonymous nature of participation, and provided instructions for obtaining the questionnaires. Interested individuals could request the materials from reception staff or their physician. Two different questionnaires were administered, one committed to the use of FS and the other on the physical and mental health (SF-12). Data collection was conducted over approximately 6–7 months to minimize disruption to routine healthcare activities.

#### 2.2.1. Questionnaire on the Use of FS

The questionnaire was prepared in clear, accessible Italian and organized into 14 items. It was arranged based on previous studies and accordingly with guidelines issued by the Italian Ministry of Health, however the questionnaire has not been formally validated [10,14,15]. The questionnaire was developed by our research team (including primary care physicians, a biologist, and a public health researcher) to assess key dimensions of FS consumption in primary care settings. However, it has not undergone formal psychometric validation. Specifically, no assessment of internal consistency (e.g., Cronbach’s alpha), test-retest reliability, construct validity, or responsiveness to change was performed. Prior to full implementation, the questionnaire underwent face validity assessment by three independent primary care physicians not involved in the study, who confirmed its clarity and clinical appropriateness. No formal pilot testing with patients was conducted. The 14 items covered the following domains: (1) FS usage patterns (current use, frequency, duration, types of supplements); (2) reasons and motivations for use; (3) sources of information and recommendation; (4) perceived effectiveness and satisfaction; and (5) concurrent health conditions and medication use. No prior formal testing was conducted. The item selection was guided by three main considerations: clinical relevance (questions were formulated based on common patterns and concerns observed in routine clinical practice regarding FS consumption), practical feasibility (items were designed to be easily administered during routine GP consultations without significantly extending appointment times), and patient comprehensibility (language and question structure were adapted to ensure accessibility for our diverse patient population). Prior to full implementation, the questionnaire underwent face validity assessment by three independent primary care physicians not involved in the study, who confirmed its clarity and clinical appropriateness. No formal pilot testing with patients was conducted. The Italian version of this questionnaire is available in Supplement Material (File S1; For English translation see File S2).

#### 2.2.2. Short Form-12 (SF-12) Questionnaire

The Italian Short Form-12 (SF-12) questionnaire [11,16] is organized into 12 items, such as physical function, role physical, social function, body pain, general health, vitality, emotional role, mental health. Higher scores can suggest better physical and mental health related quality of life, in addition to less dysfunction and impairment. Scores were calculated using online automated algorithms [17]. SF-12 was administered to the participants concurrently with the previous questionnaire. Mean Italian population scores were already calculated elsewhere [18]: PCS-12 = 50.7 and MCS-12 = 48.8. The Italian version of this questionnaire is available in Supplement Material (File S3). In this study, psychophysical well-being is operationalized as health-related quality of life (HRQoL), measured using the validated Italian version of the SF-12 questionnaire, which assesses both physical and mental health dimensions over a standard one-month recall period. This instrument was selected for its established validity, brevity, and suitability for use in primary care settings.

### 2.3. Data Handling and Quality Control

All questionnaires were reviewed manually upon collection to identify incomplete or inconsistent responses. Questionnaires with more than 10% of items missing were excluded from the analysis. For questionnaires with isolated missing items, the specific item was treated as missing and excluded from the relevant analysis, while the remaining valid responses were retained (pairwise exclusion). No imputation of missing data was performed, given the exploratory and descriptive nature of the study. To minimize the risk of duplicate participation, participants were explicitly instructed in the study notices not to complete the questionnaire more than once. As participation was anonymous, formal duplicate checking through unique identifiers was not possible; this represents an acknowledged limitation. Data from paper questionnaires were entered manually into a dedicated spreadsheet (Microsoft Excel 2019, Microsoft Corporation, Redmond, WA, USA) by a single researcher. A random sample of 20% of entries was independently verified by a second researcher to check for transcription errors. The final dataset was checked for out-of-range values and logical inconsistencies prior to statistical analysis. No personal identifying information was retained in the analytical dataset, consistent with the anonymous nature of participation.

### 2.4. Statistical Analysis

All data are expressed and presented as both median [IQR: interquartile range] and mean ± standard deviation additionally provided to facilitate comparison with published Italian population normative reference values. Non-parametric tests (Mann-Whitney and Wilcoxon Signed Rank) were selected due to the non-normal distribution of continuous variables, as assessed by the D’Agostino–Pearson omnibus test. Adjusted analyses (e.g., multivariable regression) were not feasible due to the limited sample size relative to the number of potential confounders. Specifically, variables such as age, sex, and chronic disease burden—all plausible determinants of both FS use and HRQoL—could not be simultaneously controlled for, representing a structural limitation of this pilot investigation. Applying standard events-per-variable guidelines (typically 10–15:1 ratio), the available sample was insufficient to support stable multivariable modeling without risking overfitting and unreliable parameter estimates. This limitation constrains the ability to disentangle independent associations between FS use and quality of life from potential confounding by age, sex, chronic disease burden, and other variables. GraphPad Prism software (version 10, San Diego, CA, USA) was used for statistical analysis. Differences were considered statistically significant when *p* < 0.05.

## 3. Results

### 3.1. Study Population and Clinical Data

A total of 230 questionnaires on FS use were collected, while that on SF-12 corresponded to 192. Socio-demographic information of the participants (age, gender, etc.) was collected exclusively in SF-12 questionnaire. The participants possessed a median age of 57 [41–66] (median [IQR]) (from 20 to 87 years), the male-to-female ratio was 57.4% (calculated as male/female × 100) and the median body mass index was 23.9 (21.0–27.2) kg/m^2^. All collected demographic data have been reported in Table 1.

### 3.2. Questionnaires

#### 3.2.1. Questionnaire on the Use of FS

**Figure 1:**
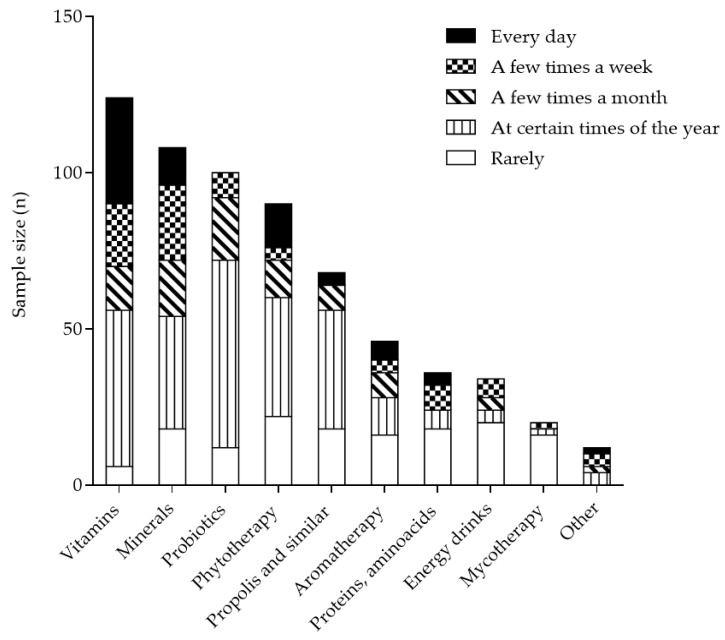
FS consumed by subjects. In black, FS consumed every day. In vertical strips, FS consumed at certain times of the year.

The SF-12 questionnaire was completed by 192 subjects (83.5% of the total sample). The remaining 38 participants (16.5%) completed only the FS questionnaire. A formal responders vs. non-responders comparison was not feasible, as sociodemographic data were collected exclusively within the SF-12 questionnaire and were therefore unavailable for non-responders. The first question addressed the type of FS used (vitamins, minerals, energy drinks, proteins and amino acids, propolis and similar, mycotherapy, aromatherapy, probiotics, phytotherapy, other) and how often they were used (every day, a few times a week, a few times a month, at certain times of the year, rarely). Bach flower remedies and homeopathic remedies were reported under the “other” category. Vitamins, minerals, and probiotics are the first three FS most selected by subjects. Furthermore, the frequency of consumption was subdivided in 5 categories: every day, a few times a week, a few times a month, at certain times of the year, and rarely. For the category “every day”, vitamins, minerals, and phytotherapy are the first three most FS consumed daily. For the category “at certain times of the year”, probiotics, vitamins, and propolis are the first three most FS selected by participants (Figure 1).

**Figure 2:**
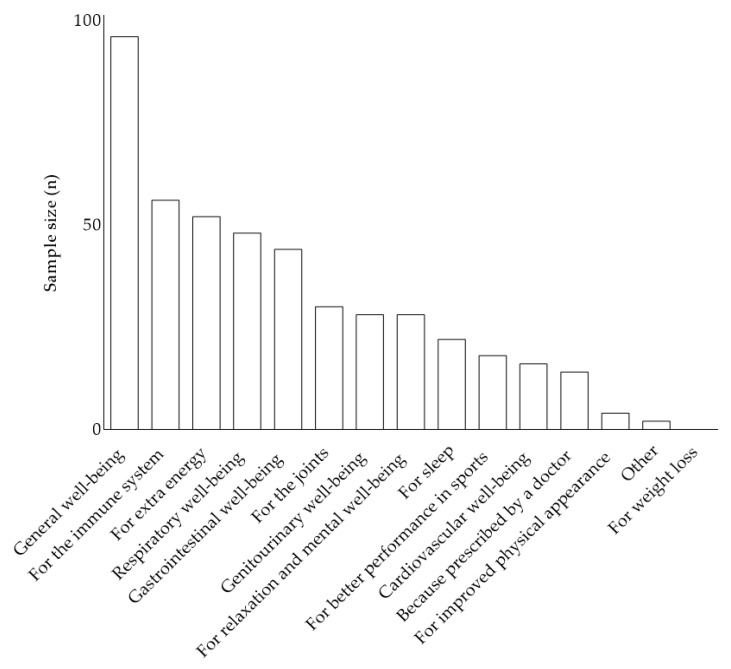
Reasons of FS use by participants.

The second question investigated why participants take a FS. Since participants could select multiple answers, percentages are calculated on the total number of responses provided (N = 458) rather than on the number of respondents. The most common answer was the use of FS for general well-being (21.0%, 96/458), followed by products for the immune system (12.2%) and for extra energy (11.4%). None of the participants reported using FS for weight loss (Figure 2).

The third question assessed the self-perceived results achieved with FS. The data showed that 16.0% of participants reported excellent results, 69.0% good results, 14.8% poor results, 0.4% no results, and 0% reported being “healed from the disorder”.

The fourth question investigated the occurrence of side effects following FS use. The results showed that 92.8% of subjects reported no side effects, while 7.3% stated psychomotor agitation, dysentery and diarrhea, hyperphagia, and insomnia.

The fifth question assessed participants’ self-perceived knowledge of the FS they were using. 53.8% of subjects reported having good knowledge about FS they were using, 37.9% poor knowledge, 3.6% no knowledge, and 4.7% excellent knowledge.

The sixth question investigated whether the participant’s GP was aware of their FS use. 57.4% of subjects confirmed that their doctor was aware, while 42.6% was not.

The seventh question asked participants whether they believed their GP would agree with their FS use. 69.8% of subjects stated that their doctor agreed, 8.3% disagreed, and 21.9% were uncertain.

The eighth question asked participants who had recommended FS to them. The results showed that GP was questioned by 34.3%, self-prescribed was chosen by 24.7%, biologist/pharmacist/healthcare worker was chosen by 17.5%, friend/colleague/family member was chosen by 16.9%, and advertising was chosen by 6.6%.

The ninth question investigated the sources from which participants obtained information about FS. 57.4% of subjects received the information from a doctor/biologist/pharmacist, 18.9% from friend/colleague/family member, 12.5% from internet, social networks and similar, 11.2% from healthcare worker (including personal trainer and excluding doctor, biologist, pharmacist).

The tenth question evaluated where participants typically purchased FS. The results showed that most purchases were made in pharmacies (55.0%), online platforms 17.7%, parapharmacy/herbalist’s shop 14.8%, supermarket 11.2%, other 1.3% (purchasing directly from the manufacturer).

The eleventh question whether the cost of FS influenced participants’ purchasing choices. The data showed that 61.7% answered affirmatively, 38.3% did not believe that the cost influenced their choice.

The twelfth question investigated in what form the participants preferred to use FS. 54.2% preferred tablets or capsules, 18.4% expressed no preference, 13.7% preferred drops, 7.2% herbal teas, and 6.5% creams, oils, or ointments.

The thirteenth question asked whether participants intended to continue using FS in the future. All subjects (100%) replied positively (no negative answer).

The fourteenth question asked which drugs were taken regularly. This question was asked to both those consumed supplements (N = 169) and those did not (N = 61). Only drugs for chronic diseases were considered, while those for acute conditions were excluded. 56.5% of subjects reported regular medication use, while 43.5% did not. A summary categorization was chosen: (a) cardiovascular drugs category (44.2%) included antiarrhythmics, antihypertensives, cholesterol-lowering drugs, etc; (b) digestive system category (14.1%) included antacids, proton pump inhibitors, intestinal anti-inflammatories, etc; (c) nervous system category (12.7%) included antidepressants, sleeping pills, antimigraine drugs, painkillers, etc; (d) hormonal system category (10.3%) included drugs for thyroid disorders, diabetes, adrenal dysfunction, etc; (e) respiratory system category (9.3%) included anti-asthmatic drugs, mucolytics, etc; (f) urogenital system category (7.9%) included estrogen-progestin drugs, anti-prostatic hypertrophy drugs, etc; (g) anti-cancer drugs (1.5%) included chemotherapy, immunotherapeutics, targeted therapy, etc.

**Figure 3:**
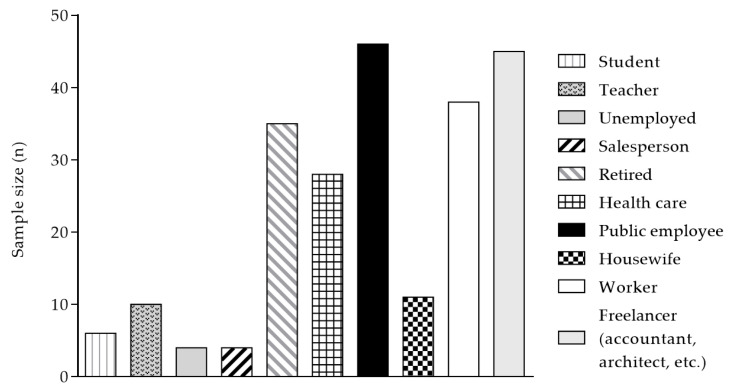
Primary occupation of participants.

The fifteenth question asked participants to report any diseases or chronic conditions they had been diagnosed with. This question was asked both to those consumed FS (N = 169) and those did not (N = 61). Only chronic diseases were considered, while acute conditions were excluded. 63.2% reported to be affected by a chronic condition, 36.8% reported not. A summary categorization was chosen: (a) cardiovascular diseases (41.6%); (b) hormonal system diseases (15.3%); (c) nervous system diseases (12.3%); (d) digestive system diseases (12.0%); respiratory system diseases (11.1%); urogenital system diseases (6.2%); cancer (1.5%). The sixteenth question asked participants to identify their primary occupation (e.g., factory worker, clerk, etc.). The results are summarized in Figure 3.

The seventeenth question was limited to participants who stated at the beginning of the questionnaire that they did not use FS (N = 61). 37.7% reported that they “didn’t feel the need for FS”, 17.4% reported price too high/non-refundable, 13.4% reported lack of adequate information, 13.4% lack of confidence, 8.2% because “it was not suggested by GP”, 6.6% reported uselessness, 3.3% reported side effects.

#### 3.2.2. Short Form-12 (SF-12) Questionnaire

The SF-12 questionnaire was completed by 192 subjects, representing 83.5% of the total sample (N = 230). Therefore, 38 participants did not complete the questionnaire on mental and physical well-being. As described in the Section 2, completing the SF-12 questionnaire yielded two standardized scores: PCS-12 (Physical Component Summary) and MCS-12 (Mental Component Summary), shown in Table 2.

Analysing the “FS use” and “no FS use” subgroups separately, subjects who declared taking FS (N = 131) had a median PCS-12 score of 50.90599 [IQR: 42.32907–55.21909] and a median MCS-12 score of 47.35788 [IQR: 39.56951–53.22978], while subjects who did not use FS (N = 61) reported a median PCS-12 of 53.64955 [IQR: 39.27472–55.77918] and a median MCS-12 of 48.13660 [IQR: 39.72620–52.84131]. Statistical comparison between the two subgroups revealed no significant difference in either the physical score (PCS-12: *p* = 0.8417) or the mental score (MCS-12: *p* = 0.9744) (Mann-Whitney test). A comparison was also made with the theoretical mean of the general Italian population (PCS-12 = 50.7 and MCS-12 = 48.8) [18]. Applying the one-sample Wilcoxon Signed Rank test to compare the sample distribution against the Italian population reference values (PCS-12 = 50.7 and MCS-12 = 48.8), the sample scores did not differ significantly from the reference for PCS-12 (median 51.22 [IQR: 41.58–55.45], mean 48.76 ± 8.43; *p* = 0.0511), while the MCS-12 score was significantly lower than the reference value (median 47.36 [IQR: 39.79–52.82], mean 45.64 ± 9.76; *p* = 0.0024). However, this mental health decrement characterized the entire GP-attending sample rather than being specifically associated with FS use or non-use.

## 4. Discussion

This work examined the general consumption of FS and perceived mental and physical well-being among a sample population attending GP offices in the Veneto region. This was a descriptive and exploratory pilot study with no a priori hypotheses regarding associations between FS use and health outcomes. The research collected 230 questionnaires on FS use and 192 on mental and physical well-being. The study population had a median age of 57 years [IQR: 41–66], a median weight of 70 kg [IQR: 56–82], and a median height of 167 cm [IQR: 160–175] (Table 1), aligning closely with the average Italian population demographics [19]. The predominance of female participants (63.5%) is consistent with national data indicating higher FS consumption among Italian women [8]. This pattern reflects gender differences in health-seeking behavior, preventive health engagement, and supplement type preferences, with women favoring multivitamins, minerals, and probiotics for general well-being, while men more commonly use protein-based or sport-oriented supplements. Life-stage specific needs, such as those related to reproductive health or bone density, may further contribute to elevated supplement use among women.

Participants demonstrated high levels of cooperation, possible facilitated by the waiting room setting, which provided ample time for questionnaire completion. Of the 230 subjects who completed the FS questionnaire, 73.5% reported using FS. This finding aligns with previous research, notably the Eurispes 2023 Report, which indicated that 68.5% of the Italian population consumed dietary supplements, with varying administration patterns [20]. While the Eurispes Report showed 14.4% regular users and 54.1% occasional users, our study found a higher proportion of daily FS users at 33% (76/230), potentially reflecting increased consumption trends or other unidentified factors. Interestingly, our findings on FS consumption (73.5%) closely mirror a recent study among Italian university students [12], which reported 71.5% FS use, despite significant demographic differences (mean age 22.5 ± 3.7 vs. median age 57 years [IQR: 41–66] in our study). The questionnaire’s structure made it challenging to precisely quantify occasional FS intake, as participants could select multiple options for non-regular use. A primary finding of this study is that FS consumption is widespread among patients attending GP offices in the Veneto region, consistent with national data, a trend likely to carry significant health implications as noted elsewhere [5,21]. Given these findings, it would be prudent for GPs to routinely consider FS use among their patients, taking into account potential interactions with prescribed drugs or other therapies. In addition, the FS questionnaire revealed that the primary reason for supplement use was “general well-being,” accounting for approximately 20% of all responses (Figure 2). This deliberately ambiguous term suggests that participants were seeking a mean to address daily challenges [8], aligning with Gallè and collaborators findings where university students reported using FS for general health [12]. The second most common reason, “for immune health” (12%), could hypothetically reflect heightened awareness of infectious diseases following the COVID-19 pandemic [22], though this interpretation was not directly tested in this study and should be regarded as speculative. “For more energy” ranked third (11%): while it is tempting to interpret this finding within the context of broader social pressures demanding greater performance [23], this study did not collect data on occupational stress or sleep patterns, and such an interpretation remains speculative. Moreover, results from the third question demonstrated high satisfaction levels among FS users, with 84.6% reporting either “excellent” or “good” experiences (27 + 116 out of 169 respondents). Only 15.4% reported negative experiences (“poor” or “none”), further corroborating the increased FS usage among the Italian population [5,20,21]. Notably, 12 out of 166 respondents (7.2%) reported experiencing side effects, a rate that warrants careful consideration. Italy has implemented the VigiErbe system (www.vigierbe.it (accessed on 22 April 2026)), a surveillance portal managed by the Istituto Superiore di Sanità and accessible to both healthcare professionals and the general public [24]. Since 2002, this phytosurveillance system has been collecting spontaneous reports of suspected adverse reactions following the consumption of dietary supplements, herbal products, magistral preparations (including those based on medical cannabis), unregistered homeopathic medicines, and other naturally-derived products. The system has documented 2700 reports since its inception, averaging approximately 117 reports annually [24]. While a direct comparison between our results and VigiErbe data is not feasible, these findings should prompt reflection on the importance of monitoring adverse events to safeguard public health. Interestingly, the thirteenth question revealed a 100% willingness among participants to purchase FS, suggesting a focus on perceived safety while potentially overlooking possible interactions and the misconception that all natural products are inherently harmless [25]. This highlights the need for improved consumer education regarding the proper use and potential risks associated with FS. The sixth question assessed whether participants informed their GP about FS use. While 54.4% of subjects reported doing so, a significant 43.6% had not, potentially leaving GPs with an incomplete picture of their patients’ health status. This low rate of patient-physician communication regarding FS use, despite participants being surveyed within a clinical setting, represents a significant patient safety concern. The combination of high self-prescription rates (66.3%) and concurrent regular medication use (56.5%) creates conditions where clinically relevant drug-supplement interactions may go undetected. Contributing factors include patients’ perception of FS as outside the medical domain, reluctance to disclose use due to anticipated physician disapproval, and lack of systematic inquiry by GPs during routine consultations. As above suggested, GPs should routinely ask patients about FS use as part of medication reconciliation, and health communication strategies should actively encourage patients to disclose all supplement use to their healthcare providers. These empirical findings—specifically the high self-prescription rate (66.3%), the low prevalence of reported side effects (7.3%), and the widespread perception of FS as safe or beneficial (84.6% reporting good or excellent results)—can be coherently interpreted through established theoretical frameworks. Risk perception theory documents a systematic tendency to underestimate risks associated with products labeled as “natural” [26], a cognitive bias termed the “naturalistic fallacy”, whereby individuals conflate botanical or natural origins with inherent harmlessness, even in the absence of rigorous safety evidence [27,28]. This bias plausibly explains why 92.8% of participants reported no side effects and why a large proportion felt confident self-prescribing without professional consultation. The Health Belief Model further contextualizes these patterns, suggesting that FS adoption reflects low perceived susceptibility to natural product-related harm, high perceived severity of conventional drug side effects, and low barriers to over-the-counter access [29]. Critically, these theoretical frameworks suggest that effective clinical communication must go beyond simply providing factual risk information, since the natural-equals-safe heuristic operates at a pre-rational, emotion-driven level that is resistant to straightforward educational interventions [25]. This has direct implications for how GPs approach conversations about FS with their patients, emphasizing the need for targeted communication strategies that address underlying affective associations rather than relying solely on information provision. The fifth question further revealed that 58.6% of participants considered themselves well-informed about FS (“good” and “excellent” responses), compared to 41.4% who declared “little” or “no” knowledge. This self-perceived understanding is concerning, as it may indicate a false sense of security regarding FS, potentially leading to the misconception that “natural equates to harmless” a problem previously encountered in complementary medicine [30,31]. Concurrently, GP often lack comprehensive knowledge about FS adverse effects, a topic largely neglected in Italian medical education [32]. Addressing this knowledge gap through improved, quality information is crucial, as previously suggested [32]. The seventh question revealed that approximately 70% of patients perceived their GP as agreeing with their FS use, demonstrating GPs’ openness and willingness to accommodate patients’ concerns. However, 30% felt their GP disagreed, possibly due to perceived opposition to FS use, beliefs about exclusive medical authority, or other undetermined factors. Notably, the eighth question showed that professional advice affected FS use in 50.9% of cases. While this indicates that half of the patients received guidance from a professional, it also highlights that the remaining participants relied on advice from friends, relatives, advertising, or self-prescription when choosing FS. Interestingly, these findings appear to diverge from the CENSIS report, which suggested that healthcare professionals serve as the primary point of reference for FS advice, with an estimated 82.4% of Italian FS users receiving recommendations from GPs, specialists, or pharmacists [5]. This discrepancy highlights potential shifts in consumer behavior or regional variations in FS consultation patterns. The contrast between our results and the CENSIS data underscores the need for further investigation into FS usage patterns and information-seeking behaviors. It may indicate evolving trends in how consumers approach FS use, possibly reflecting changes in information accessibility, marketing strategies, or trust in various information sources. This discrepancy also emphasizes the importance of continued efforts to ensure that accurate, professional advice reaches FS users, potentially through improved communication channels between healthcare providers and patients. In addition, the excessive use of these products appears to be a uniquely Italian phenomenon, with Italy leading the European market with a 27% share [5]. This indirectly emphasises a lack of awareness in their use, probably influenced by advertising and the common misconception that “natural means good and safe” [30,31]. The ninth question aimed to understand where participants obtained information on FS use. While 57.4% sought information from professional sources (GPs, biologists, pharmacists, etc.), a significant 42.6% relied on unofficial or less reputable channels. This finding, strictly connected with eighth question and its results, raises concerns and suggests that subjects often bypass professional advice, particularly from their GPs. The tenth question revealed that pharmacies remained the most popular place to purchase FS, accounting for over 50% of sales. However, this figure appears lower compared to the 76.3% reported by Censis in 2019, suggesting a potential shift towards other channels, particularly online platforms, over the past six years [5]. Related to this, the eleventh question showed that 61.7% of subjects believed the cost of FS could affect their purchasing choices. This price sensitivity may contribute to the growing use of online channels or large supermarkets offering health product sections, where FS are often available at lower prices. Questions regarding medication use and self-reported diseases revealed a clinically complex picture. As expected, the most common drugs and pathologies were related to the cardiovascular system, aligning with its status as the leading cause of death worldwide [33]. As above mentioned, these findings underscore the need for improved consumer education, more robust regulation of FS marketing and sales channels, and enhanced communication between healthcare providers and patients to ensure safe and informed use of dietary supplements. Due to the limited sample size, further statistical correlations (e.g., between occupation category and specific FS use, or between chronic disease burden and specific FS use) were not feasible. Similarly, while a predominance of employees and freelancers was observed among participants, the sample size precluded meaningful statistical correlation of occupational data with other study variables. The final question of FS questionnaire, completed exclusively by non-FS users (26.5% of respondents), revealed that the primary reason for not using FS was a perceived lack of need (37.7%). This result supports the hypothesis that these individuals may maintain a healthy lifestyle, already undergo pharmacological treatments, or use other therapeutic approaches. This observation underscores the importance of considering individual health status and lifestyle factors when evaluating FS use patterns in the population.

The SF-12 questionnaire identified two scores: the PCS-12 (Physical Component Summary) and the MCS-12 (Mental Component Summary). A central finding of this exploratory study was the absence of significant associations between FS use and health-related quality of life. However, three substantial methodological limitations must be explicitly acknowledged. First, the study was underpowered: with a sample of 192 participants and no a priori power calculation, the study may have lacked sufficient statistical power to detect small or moderate effect sizes. Second, confounder adjustment was not feasible: key variables such as age, sex, and chronic disease burden—all plausible determinants of both FS use and HRQoL—could not be simultaneously controlled for. Third, a temporal mismatch exists between instruments and this discordance is particularly relevant for seasonal FS users. Collectively, these constraints mean that the null finding should be interpreted as hypothesis-generating. This null finding indicates that FS consumption was not associated with better or worse physical or mental health status in this sample. The independence of quality of life from FS use suggests that individuals choosing FS do not constitute a distinct health subpopulation in terms of functional status or well-being. These results should be interpreted cautiously given the cross-sectional design (i.e., data were collected at a single point in time, which does not allow conclusions about cause-and-effect relationships), which precludes assessment of whether FS use influences quality of life trajectories over time, and the exploratory nature of this investigation. Furthermore, analysis revealed that the MCS-12 score was significantly lower than the Italian population reference value, indicating a poorer perception of mental well-being among the study participants. Specifically, this suggests that the sample (N = 192) may have experienced alterations in mood, energy levels, and symptoms of anxiety and depression in the four weeks preceding the questionnaire. This finding was likely influenced by selection bias, as participants were visiting their GP’s office and were therefore presumably experiencing some degree of health-related need. These subjects represent a specific subpopulation that differ systematically from the general population and could have affected several key outcomes, including the lower MCS-12 mental health scores observed (this may reflect the psychological burden associated with illness or healthcare-seeking behavior rather than a true population-level trend). Consequently, a decline in perceived mental well-being could be expected. In contrast, the PCS-12 value did not deviate significantly from the population reference value, which is unexpected given that people typically visit doctors for physical health concerns rather than psychological issues. Critically, these quality of life patterns were independent of FS use: the observed mental health decrements characterize the GP-attending sample broadly rather than being associated with FS consumption. Several hypotheses could explain these results. In order, (a) selection bias: the study population may have consisted of individuals with sufficient mobility and relatively preserved physical health, capable of visiting their GP and waiting in the office. Those with more severe conditions may have been unable to attend; (b) altered self-perception of physical health: one possible hypothesis is that patients undergoing pharmacological treatment may perceive their physical health more positively due to symptom management, even if underlying conditions persist, a phenomenon sometimes termed “treatment-induced optimism” [34]. However, this interpretation was not directly tested in this study and should be considered a speculative explanation for the observed PCS-12 findings rather than a data-supported conclusion. However, such treatments may not comparably improve psychological well-being, leaving MCS-12 scores reflective of true mental health burden; (c) pandemic-related psychological impact: the observed reduction in MCS-12 scores may partly reflect the well-documented increase in psychological distress in the post-pandemic period [35], though this remains a hypothetical interpretation not assessed in this study. These findings are consistent with the hypothesis that primary care attendees may experience greater psychological burden compared to the general population, possibly reflecting the stress associated with illness and healthcare-seeking behavior. Yet, since psychological vulnerability was not directly measured in this study, this interpretation should be regarded as a purely plausible hypothesis. Importantly, this mental health burden is not attributable to FS use but rather reflects broader characteristics of individuals seeking primary care. Clinicians should maintain awareness of mental health needs in ambulatory patients, regardless of presenting complaints. The absence of statistically significant differences in HRQoL between FS users and non-users should not be interpreted as definitive evidence that FS consumption has no effect on health status. These findings should therefore be regarded as preliminary and hypothesis-generating, requiring confirmation in larger, adequately powered studies with more targeted outcome measures.

The study presented some limitations. First, waiting-room recruitment introduces selection bias, as GP attendees differ systematically from the general population, limiting generalizability. Second, since participants self-selected, the total number of individuals exposed to study materials is unknown, precluding calculation of a formal response rate and assessment of non-response bias. Third, all data were collected via self-report without objective verification, introducing potential information bias and social desirability effects, particularly regarding self-prescription and physician communication. Fourth, the FS questionnaire was not formally psychometrically validated, meaning measurement error cannot be quantified and findings should be interpreted accordingly. In addition, not all participants completed the questionnaire correctly, sometimes missing some answers or skipping one of the two questionnaires. Consequently, a direct numerical correspondence between the FS questionnaire and SF-12 completion rates could not be established. In addition, the limited sample size represents a significant constraint on the generalizability of findings. This limitation is particularly relevant when interpreting the null associations observed between FS use and health-related quality of life, as the study may have lacked sufficient statistical power to detect small or moderate effect sizes. Although this study represents a novel contribution to the field and recruited participants from multiple geographic areas across the Veneto region, the cohort cannot be considered representative of the broader regional population. Moreover, excluding participants unable to complete questionnaires (exclusion of individuals unable to correctly complete the questionnaires), while justified for data quality, could introduce a “healthy responder bias” with possible overestimation of physical health scores (PCS-12) that can explain why PCS-12 scores didn’t differ significantly from Italian population norms. A methodological consideration concerns the divergent temporal frames of reference: the FS questionnaire assessed efficacy over 12 months, while the SF-12 employed a standard one-month recall period. This temporal discordance has important implications for interpreting our null findings. If FS effects manifest gradually, the one-month SF-12 window may not capture them. Conversely, seasonal FS use patterns (reported by participants as “at certain times of the year”) mean some users may have been assessed during non-use periods, potentially masking true associations. Fundamentally, we are correlating long-term behavior with short-term functional status, which precludes causal inference. Extension of the SF-12 recall period was not feasible, as this would compromise the instrument’s validated psychometric properties [17]. While acceptable for this exploratory descriptive study, these temporal considerations reinforce that null associations should be interpreted descriptively rather than as evidence for or against causal relationships. However, since these questionnaires assess fundamentally different domains (disease-specific efficacy versus generic health-related quality of life), perfect temporal congruence was not considered a critical requirement for the study objectives and any findings should be interpreted descriptively rather than causally. The use of a non-validated questionnaire represents a significant methodological limitation, as it may compromise the reliability and accuracy of the findings. The absence of formal psychometric testing means that measurement error cannot be quantified, and the instrument’s ability to consistently and accurately capture FS consumption patterns is uncertain. Future studies should prioritize the development and validation of standardized tools for this purpose. An important caveat is that the FS questionnaire employed in this study has not been subjected to formal psychometric validation (e.g., reliability, validity, responsiveness), and it is currently available only in Italian. These limitations constrain the instrument’s applicability to non-Italian populations and hinder direct comparison with international studies. Future research should prioritize the validation of this tool and its translation into other languages to facilitate broader implementation. A relevant limitation concerns selection bias inherent to the recruitment setting. Patients attending GP offices may differ from the general population in terms of health status, health-seeking behavior, and supplement use awareness. Those with more severe or limiting conditions may have been unable to attend, while healthier individuals may have been underrepresented. Consequently, the observed prevalence of FS use and the patterns identified may not be fully generalizable to the broader regional or national population.

Furthermore, the absence of adjusted analyses limits our ability to account for potential confounders (age, sex, chronic disease burden) that may influence both FS use and quality of life. As a result, observed associations—or the absence thereof—between FS use and HRQoL may be confounded by these unmeasured or unadjusted factors. Without multivariable adjustment, it is impossible to determine whether observed patterns reflect independent associations or are mediated by these factors. This further reinforces the need to interpret all findings descriptively rather than as evidence of causal or independent associations. The null associations observed between FS use and HRQoL should be interpreted with considerable caution. In the absence of multivariable adjustment, it is not possible to determine whether the observed patterns reflect true independence or are instead confounded by variables such as age, chronic disease burden, or sex, all of which may independently influence both FS use and quality of life.

## 5. Conclusions

This pilot study demonstrates that FS use is common among GP attendees in the Veneto region, is primarily motivated by general well-being, and is not always fully communicated to physicians. No association between FS use and SF-12 scores was detected in this sample. These findings should be regarded as descriptive observations from an exploratory pilot study rather than definitive conclusions; whether FS use influences clinical health outcomes, and which patient and contextual factors mediate GP communication about FS, remain open questions requiring larger, longitudinal, and methodologically more robust investigations. In addition, this work forms part of a broader initiative to promote awareness and informed use of FS among both GPs and patients. The research serves as a crucial first step towards a comprehensive educational objective, drawing attention to the fact that while FS are not classified as drugs, they can potentially interact with medications and may have side effects. By highlighting these aspects, the study seeks to foster a more nuanced understanding of FS, encouraging their responsible use and integration into healthcare practices. Future research with larger, representative samples and longitudinal designs is needed to definitively assess whether FS use influences health outcomes over time.

## Figures and Tables

**Table 1:** Anthropological data.

	Median (IQR)	Mean ± SD	Confidence Interval 95%		Maximum Value	Minimum Value
			Lower	Highest	-	-
Age (year)	57 (41–66)	53.6 ± 17.6	51	56	87	20
Male (N)	70	-	-	-	-	-
Female (N)	122	-	-	-	-	-
Height (cm)	167 (160–175)	168.0 ± 9.7	167	170	193	150
Weight (kg)	70 (56–82)	70.3 ± 16.0	68	73	125	46
BMI (kg/m^2^)	23.9 (21.0–27.2)	24.6 ± 4.3	24.0	25.2	38.1	17.4

**Table 2:** PCS-12 and MSC-12 values.

	PCS-12 (Median [IQR])	MSC-12 (Median [IQR])	PCS-12 (Mean ± SD)	MSC-12 (Mean ± SD)
Subjects (N = 192)	51.21875 [41.58251–55.45119]	47.35788 [39.78632–52.81556]	48.75996 ± 8.431984	45.64293 ± 9.758459
Minimum values	26.50734	20.76708	26.50734	20.76708
Maximum values	61.71383	59.89161	61.71383	59.89161

## Data Availability

The data presented in this study are available on request from the corresponding author. Data collected from all subjects are unavailable due to privacy restrictions.

## Supplementary Materials

The following supporting information can be downloaded at: https://www.mdpi.com/article/10.3390/healthcare14091189/s1, File S1: QUESTIONNAIRE on the use of FOOD SUPPLEMENTS; File S2: QUESTIONNAIRE on the use of FOOD SUPPLEMENTS translated in English; File S3: Health status questionnaire.

## Abbreviations

The following abbreviations are used in this manuscript:

FS	Food supplement
GP	General practitioner
MCS-12	Mental Component Summary
PCS-12	Physical Component Summary
