# Supplementary material for: Food Supplements and Well-Being: A Pilot Investigation in the General Practitioner Office of the Veneto Region
Source: Healthcare (Basel). 2026 Apr 29;14(9):1189. doi: 10.3390/healthcare14091189 (PMC13164215; doi:10.3390/healthcare14091189)
Supplement: Supplementary file 1 [file healthcare-14-01189-s001.zip › supplement material S1.pdf]

# QUESTIONARIO sull'uso di INTEGRATORI ALIMENTARI

Il questionario durerà all'incirca 10 minuti. Accetta di partecipare al questionario rispondendo alle successive domande? SI NO ⟶ **FINE QUESTIONARIO**

Riferirsi al consumo di integratori alimentari negli ultimi 12 mesi (quindi comprensivo del periodo invernale). **Gli integratori alimentari NON sono farmaci, alcol, droghe o qualsiasi alimento che fa parte della normale dieta**. Gli integratori sono ad esempio VITAMINE, SALI MINERALI, PROTEINE, AMINOACIDI, ACIDI GRASSI ESSENZIALI, FIBRE, ENERGY DRINK, PIANTE, PROBIOTICI, ECC.

## *Consuma integratori?* SI NO ⟶ *vai alla domanda 14*

*1. Quali integratori consuma?*

| **Tipo di integratore** | **Quante volte li usa?** | | | | |
|---|---|---|---|---|---|
| | Tutti i giorni | Poche volte a settimana | Poche volte al mese | In determinati periodi dell'anno | Raramente |
| Vitamine (da sole o in combinazione) | | | | | |
| Sali minerali (da soli o in combinazione) | | | | | |
| Energy drink | | | | | |
| Proteine, aminoacidi e simili | | | | | |
| Propoli o altri prodotti delle api | | | | | |
| Micoterapia (funghi) | | | | | |
| Aromaterapia (oli essenziali) | | | | | |
| Probiotici (fermenti lattici) o prebiotici | | | | | |
| Prodotti naturali o derivati da piante. Specificare: | | | | | |
| Altro (specificare): | | | | | |

*2. Perché assume gli integratori (possibili più risposte)?*

- o Per il benessere generale
- o Per il benessere cardio-circolatorio (ad esempio per il colesterolo)
- o Per il benessere gastro-intestinale (ad esempio per la stipsi)
- o Per il benessere genito-urinario (ad esempio per la cistite)
- o Per il benessere respiratorio e gola (ad esempio per il raffreddore)
- o Per le articolazioni
- o Per il sistema immunitario e combattere le infezioni
- o Per il sonno
- o Per il relax e il benessere mentale
- o Per essere più performante nello sport
- o Per avere più energia
- o Per perdere peso
- o Per migliorare l'aspetto fisico
- o Perché prescritto dal medico
- o Altro (specificare):___________________

*3. Quali risultati ha ottenuto con gli integratori assunti?*
- ottimi - buoni - scarsi - nessuno
- guarito dal disturbo

*4. Ha notato degli effetti collaterali a seguito dell'assunzione dell'integratore/i?*
NO
SI quali? ___________________________
_____________________________________

*5. Qual è la sua conoscenza degli integratori che assume?*
- ottima - buona - scarsa - nessuna

*6. Il suo medico è a conoscenza del fatto che assume un integratore/i?*
SI NO

*7. Crede che Il suo medico sarebbe d'accordo sul fatto che lei assuma un integratore/i?*
SI NO NON SO

*8. Chi le ha consigliato l'integratore/i?*
- o Medico
- o Biologo/dietista/nutrizionista
- o Amico/collega/famigliare
- o Pubblicità
- o Auto-prescritto
- o Altro (specificare):________________

*9. Da dove ha ottenuto informazioni per l'assunzione dell'integratore/i?*
- o Professionista (medico, biologo, farmacista)
- o Amici, famigliari, conoscenti
- o Personal trainer
- o Operatore sanitario
- o Internet, social network
- o Altro (specificare):________________

*10. Dove acquista normalmente l'integratore/i?*
- o farmacia
- o parafarmacia/erboristeria
- o supermercato
- o online
- o Altro (specificare):________________

*11. Ritiene che il costo dell'integratore influenzi la sua scelta?*
SI NO

*12. Sotto quale forma preferisce usarli?*
- compresse - gocce - tisane - oli
- creme/pomate - altro (specificare)

*13. Consumerà ancora integratori in futuro?*
SI NO

*14. Assume regolarmente farmaci? Quali?*
___________________________________
___________________________________
___________________________________

*15. Di quali malattie soffre (ad es. diabete, ipertensione, ecc.)?*
___________________________________
___________________________________
___________________________________

*16. Che lavoro sta facendo o ha fatto principalmente (ad es. operaio, impiegato, ecc.)?*
___________________________________
___________________________________

---- ---- ---- ---- ----

**17. SOLO PER CHI HA RISPOSTO NO ALL'INIZIO DEL QUESTIONARIO**
*Per quale motivo non assume integratori (possibile più risposte)?*
- o Inutilità/ sconsigliato da esperti
- o Non fiducia
- o Effetti collaterali
- o Mancanza di informazioni adeguate
- o Prezzo troppo alto / non rimborsabili
- o Altro (specificare): _______________
___________________________________

**FINE QUESTIONARIO, GRAZIE DELLA COLLABORAZIONE.**

***Consideri di condividere le informazioni sull'uso degli integratori con il suo medico, certamente lo apprezzerà così come la sua salute!***
