# Supplementary material for: Food Supplements and Well-Being: A Pilot Investigation in the General Practitioner Office of the Veneto Region
Source: Healthcare (Basel). 2026 Apr 29;14(9):1189. doi: 10.3390/healthcare14091189 (PMC13164215; doi:10.3390/healthcare14091189)
Supplement: Supplementary file 1 [file healthcare-14-01189-s001.zip › supplement material S2.pdf]

QUESTIONNAIRE on the use of FOOD SUPPLEMENTS

The questionnaire will take approximately 10 minutes. Do you agree to participate by answering the following questions

SI NO → **END of QUESTIONNAIRE**

Food supplements are NOT medicines, alcohol, drugs, or any food that is part of a normal diet. Supplements include, for example: VITAMINS, MINERALS, PROTEINS, AMINO ACIDS, ESSENTIAL FATTY ACIDS, FIBRE, ENERGY DRINKS, PLANTS, PROBIOTICS, ETC.

Do you consume supplements? SI NO → ***go to question 14***

1. Which supplements do you take?

**Type of supplement**	**How often do you use them?**				
	Every day	A few times a week	A few times a month	At certain times of the year	Rarely
Vitamins (alone or in combination)					
Minerals (alone or in combination)					
Energy drinks					
Proteins, amino acids and similar					
Propolis or other bee products					
Mycotherapy (mushrooms)					
Aromatherapy (essential oils)					
Probiotics (lactic acid bacteria) or prebiotics					
Natural or plant-derived products. Specify:					
Other (specify):					

2. Why do you take supplements? (multiple answers possible)

For general wellbeing
For cardiovascular health (e.g. cholesterol)
For gastrointestinal health (e.g. constipation)
For genitourinary health (e.g. cystitis)
For respiratory health and throat (e.g. colds)
For joints
For the immune system and fighting infections
For sleep
For relaxation and mental wellbeing
To improve sports performance
To have more energy
To lose weight
To improve physical appearance
Because prescribed by a doctor
Other (specify): ________________

3. What results have you achieved with the supplements taken?
Excellent – Good – Poor – None – Recovered from the condition

4. Have you noticed any side effects following the use of the supplement(s)?
NO
SI Which ones?____________________
____

5. How would you rate your knowledge of the supplements you take?
Excellent – Good – Poor – None

6. Is your doctor aware that you take supplement(s)?
SI NO

7. Do you think your doctor would agree with you taking supplement(s)?
SI NO NON SO

8. Who recommended the supplement(s) to you?
Doctor
Biologist/dietitian/nutritionist
Friend/colleague/family member
Advertising
Self-prescribed
Other (specify): _______________

9. Where did you get information about taking the supplement(s)?
Professional (doctor, biologist, pharmacist)
Friends, family, acquaintances
Personal trainer
Healthcare worker
Internet, social networks
Other (specify): _______________

10. Where do you usually purchase the supplement(s)?
Pharmacy
Parapharmacy/herbalist's shop
Supermarket
Online
Other (specify): _______________

11. Do you think the cost of the supplement influences your choice?
SI NO

12. In what form do you prefer to use them?
Tablets – Drops – Herbal teas – Oils – Creams/ointments – Other (specify)

13. Will you continue to use supplements in the future?
SI NO

14. Do you regularly take medicines? Which ones?
____
____
____

15. What conditions do you suffer from (e.g. diabetes, hypertension, etc.)?
____
____
____

16. What is or was your main occupation (e.g. factory worker, office worker, etc.)?
____
____

---- ---- ---- ---- ----

17. ONLY FOR THOSE WHO ANSWERED NO AT THE BEGINNING
Why don't you take supplements?
(multiple answers possible)
Uselessness / advised against by experts
Lack of trust
Side effects
Lack of adequate information
Too expensive / not reimbursable
Other (specify): _______________

END OF QUESTIONNAIRE, THANK YOU FOR YOUR PARTICIPATION.
Consider sharing information about your supplement use with your doctor — they will certainly appreciate it, and so will your health!
