# Supplementary material for: Food Supplements and Well-Being: A Pilot Investigation in the General Practitioner Office of the Veneto Region
Source: Healthcare (Basel). 2026 Apr 29;14(9):1189. doi: 10.3390/healthcare14091189 (PMC13164215; doi:10.3390/healthcare14091189)
Supplement: Supplementary file 1 [file healthcare-14-01189-s001.zip › supplement material S3.pdf]

SF-12

Questionario sullo stato di salute

Data di nascita **Sesso** F M **Data di oggi**

1. **In generale, direbbe che la Sua salute è**

   Eccellente.……................. 1 ☐

   Molto buona................................. 2 ☐

   Buona.………..................................... 3 ☐

   Passabile.. 4 ☐

   Scadente... 5 ☐

2. **La sua salute La limita attualmente nello svolgimento di attività di moderato impegno fisico (come spostare un tavolo, usare l'aspirapolvere, giocare a bocce o fare un giro in bicicletta, ecc.) ?**

   SI, mi limita parecchio…………. 1 ☐

   SI, mi limita parzialmente……………. 2 ☐

   NO, non mi limita per nulla..………………. 3 ☐

3. **La sua salute La limita attualmente nel salire qualche piano di scale ?**

   SI, mi limita parecchio…………. 1 ☐

   SI, mi limita parzialmente……………. 2 ☐

   NO, non mi limita per nulla..………………. 3 ☐

4. **Nelle ultime 4 settimane, ha reso meno di quanto avrebbe voluto sul lavoro o nelle altre attività quotidiane, a causa della sua salute fisica?**

   SI…………. 1 ☐

   NO………………. 2 ☐

5. **Nelle ultime 4 settimane, ha dovuto limitare alcuni tipi di lavoro o di altre attività, a causa della sua salute fisica ?**

   SI…………. 1 ☐

   NO ………………… 2 ☐

6. **Nelle ultime 4 settimane, ha reso meno di quanto avrebbe voluto sul lavoro o nelle altre attività quotidiane, a causa del suo stato emotivo (quale il sentirsi depresso o ansioso) ?**

   SI…………. 1 ☐

   NO ………………… 2 ☐

7. **Nelle ultime 4 settimane, ha avuto un calo di concentrazione sul lavoro o nelle altre attività quotidiane, a causa del suo stato emotivo (quale il sentirsi depresso o ansioso)?**

   SI…………. 1 ☐

   NO………………. 2 ☐

8. **Nelle ultime 4 settimane, in che misura il dolore l'ha ostacolata nel lavoro che svolge abitualmente (sia in casa sia fuori casa) ?**

   Per nulla…………. 1 ☐

   Molto poco……………. 2 ☐

   Un po'………..………………. 3 ☐

   Molto……………...……………. 4 ☐

   Moltissimo.….. ………………………. 5 ☐

9. **Per quanto tempo nelle ultime 4 settimane si è sentito calmo e sereno ?**

   Sempre…………. 1 ☐

   Quasi sempre………. 2 ☐

   Molto tempo……………….3 ☐

   Una parte del tempo…………. 4 ☐

   Quasi mai... ……………………..…. 5 ☐

   Mai……………………………………..…. 6 ☐

10. **Per quanto tempo nelle ultime 4 settimane si è sentito pieno di energia?**

    Sempre…………. 1 ☐

    Quasi sempre………. 2 ☐

    Molto tempo……………….3 ☐

    Una parte del tempo…………. 4 ☐

    Quasi mai…………………………..…. 5 ☐

    Mai……………………………………..…. 6 ☐

11. **Per quanto tempo nelle ultime 4 settimane si è sentito scoraggiato e triste ?**

    Sempre…………. 1 ☐

    Quasi sempre………. 2 ☐

    Molto tempo……………… 3 ☐

    Una parte del tempo…………. 4 ☐

    Quasi mai…………………………..…. 5 ☐

    Mai…………..……………………..…. 6 ☐

12. **Nelle ultime 4 settimane, per quanto tempo la Sua salute fisica o il Suo stato emotivo hanno interferito nelle sue attività sociali, in famiglia, con gli amici?**

    Sempre……… 1 ☐

    Quasi sempre………. 2 ☐

    Una parte del tempo……… 3 ☐

    Quasi mai ....4 ☐

    Mai ....5 ☐
